# Blurring the boundaries: developmental and activity-dependent determinants of neural circuits

**DOI:** 10.1016/j.tins.2013.06.006

**Published:** 2013-10

**Authors:** Verena Wolfram, Richard A. Baines

**Affiliations:** Faculty of Life Sciences, University of Manchester, Manchester, UK

## Abstract

The human brain comprises approximately 100 billion neurons that express a diverse, and often subtype-specific, set of neurotransmitters and voltage-gated ion channels. Given this enormous complexity, a fundamental question is how is this achieved? The acquisition of neurotransmitter phenotype was viewed as being set by developmental programs ‘hard wired’ into the genome. By contrast, the expression of neuron-specific ion channels was considered to be highly dynamic (i.e., ‘soft wired’) and shaped largely by activity-dependent mechanisms. Recent evidence blurs this distinction by showing that neurotransmitter phenotype can be altered by activity and that neuron type-specific ion channel expression can be set, and perhaps limited by, developmental programs. Better understanding of these early regulatory mechanisms may offer new avenues to avert the behavioral changes that are characteristic of many mental illnesses.

## Highlights

• Neurotransmitter phenotype can be altered by activity.
• Neuron type-specific ion channel expression can be set by developmental programs.
• Common mechanisms orchestrate the development of different neuronal signaling properties.
• *Drosophila* enables electrophysiology and genetics to be applied to identified neurons.

## Neurons express diverse signaling properties

Neural circuits in organisms as diverse as worms, flies, and humans exhibit remarkably similar design and developmental principles [1–3]. Circuit function depends on the concerted action of distinct classes of sensory neuron, regulatory interneuron and motor neuron. The function of each neuronal subtype is defined by its position, axon trajectory, synaptic connectivity, neurotransmitter expression, and electrophysiological properties. An important but unanswered question is how do neurons acquire subtype-specific properties? The answer undoubtedly depends on the relative contributions of both developmental programs (e.g., the type-specific transcription factor expression) and activity-dependent mechanisms.

Although the identification of the developmental and activity-dependent mechanisms that shape axon trajectory and neurotransmitter phenotypes has progressed [4–6], the same is not true for the regulation of expression of ionic currents in early embryonic neurons. Progress has been hampered by the lack of suitable model systems in which genetics and electrophysiology can be combined at the level of identifiable neurons. Recent developments in the fruit fly, *Drosophila melanogaster*, and zebrafish, *Danio rerio*, which allow such recordings, have started to yield important first clues. In this review, we consider these recent findings that directly relate to the question of ‘how to build a neural circuit’. We present an updated view of the potential contribution of differing regulatory mechanisms operative during development that determine active signaling properties of embryonic neurons.

## Neuronal specification

Neuronal specification occurs early during embryogenesis and, for simplicity, can be considered to comprise three broad processes: proliferation, migration, and differentiation. Although all of these processes are crucial for the proper formation of neural circuits, we concentrate on neuronal differentiation in this review. It is during neuronal differentiation that neurons first acquire their specific, and often unique, properties; these include axon projection, dendrite arborization, neurotransmitter specification, and ion channel expression. Until recently, the three former properties were considered to be highly stereotypic and were described as being ‘hard wired’. In support of this, many developmental transcription factors have been identified as important determinants that specify axon path-finding and neurotransmitter specification. By contrast, much less is known with regard to the developmental determinants that orchestrate ion channel gene expression. Indeed, this aspect of neuronal function has been shown to be dynamic and under extensive control of extrinsic neuronal activity [7] and activity-dependent homeostatic mechanisms [8]. Implicit in these studies is the concept that, unlike specification of neurotransmitters, the emergence of electrical properties in embryonic neurons is dependent on network activity and, as such, could be considered to be ‘soft wired’. However, the distinction between hard- and soft-wired properties is blurred by recent evidence that shows that neurotransmitter phenotype is influenced by activity and that ion channel expression can be set by subtype-specific intrinsic developmental mechanisms.

## Neurotransmitter specification

In comparison to ion channels, our understanding of the regulatory mechanisms that specify neurotransmitter expression is more comprehensive. The choice of neurotransmitter not only determines the functional modality of any given neuron, but is also critical for the functionality of the circuit to which each neuron contributes. As such, the specification of neurotransmitter phenotype is a key step for each and every neuron during embryonic development. Several studies have shown that neurotransmitters are set by neuron subtype-specific transcriptional programs and, as such, could be considered to be a ‘hard wired’ characteristic. However, as we describe here, recent experiments overturn this view by showing that the choice of neurotransmitter can be respecified by neural activity [5].

The setting of neurotransmitter phenotype through cell type-specific developmental programs could be considered consistent with both the robustness and stability of neurotransmitter expression throughout the life of a neuron. There are several considerations that strengthen such a hypothesis. First, many neuron types generally express only one classical neurotransmitter [most likely acetylcholine (ACh), glutamate, GABA, serotonin, noradrenaline (nAdr), or dopamine (Da)] and often neurons with different classical transmitters develop from distinct pools of neuronal precursors [9]. Second, the restricted number of neurotransmitters and, in many cases [e.g., ACh, Da, nAdr, or 5-hydroxytryptamine (5-HT)], the requirement of gene cassettes to produce that neurotransmitter is indicative of tight transcriptional regulation. Third, many circuits, such as sensory input circuitry or the motor network output, must maintain reliable transmission, which would be ensured by an early and stable encoding of neurotransmitter phenotype. Indeed, several studies (described below) describe genetic programs, active during development, that specify neurotransmitter expression.

### Transcription factor specification of neurotransmitter phenotype

In different phyla, neurotransmission at the peripheral neuromuscular junction (NMJ) is mediated by different neurotransmitters. For example, in the nematode *Caenorhabditis elegans*, NMJs comprise a mixture of excitatory cholinergic and inhibitory GABAergic synapses [10–13]. The excitatory NMJs of dipteran insects (i.e., *Drosophila*) and chordates (i.e., vertebrate) utilize glutamate and ACh, respectively [14–16]. The expectation that neurotransmitter expression is tightly regulated at NMJs has been partly met by the identification of transcription factors of the LIM domain and homeodomain (HD domain) families, which are differentially expressed in motor neurons, where they orchestrate the developmental decisions of which neurotransmitter to express [17–19].

**Figure 1:**
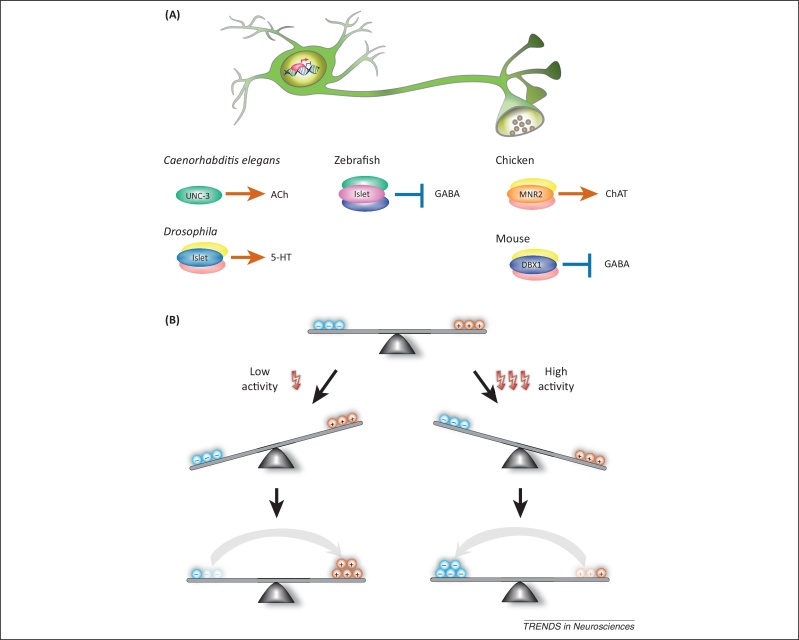
Specification of neurotransmitter phenotype. **(A)** Neurotransmitter phenotypes are genetically specified early during development by expression of developmental transcription factors. Transcription factors act by activating or repressing the transcription of proteins that are important for the synthesis and transport of neurotransmitters. Often, transcription factors are part of combinatorial codes and act together in a complex. Examples of transcription factors are given for neurons of representative species across different phyla. Arrows indicate that the transcription factors are required for expression of the respective neurotransmitter, whereas T-bars indicate a repressive effect. **(B)** Activity-dependent switching of neurotransmitter phenotypes. Studies in *Xenopus* demonstrate that enhanced (shown on the right) as well as reduced (shown on the left) neuronal activity is sufficient to induce a respecification of the neurotransmitter phenotype in neurons of the spinal cord to maintain an appropriate excitation–inhibition balance. Decreased activity favors an increase in neurons expressing excitatory neurotransmitters [acetylcholine (ACh) and glutamate, orange circles], whereas an increase in activity leads to increased numbers of GABA-expressing neurons (blue circles) [48]. Abbreviations: 5-HT, 5-hydroxytryptamine; ChAT, choline acetyltransferase; DBX1, developing brain homeobox 1; UNC-3, uncoordinated-3.

Neurotransmitter specification is arguably best understood within the eight classes of *C. elegans* motor neuron (AS, DA, DB, DD, VA, VB, VC, and VD). For example, expression of the UNC-3 transcription factor is sufficient to specify a cholinergic phenotype in type A and B motor neurons (VA, VB, DA, DB, and AS) (Figure 1A) [17], whereas the HD transcription factor UNC-30 is required for the GABAergic phenotype of D motor neurons (VD, DD) [20,21]. In addition, AST-1, an E-twenty six (ETS) domain transcription factor, is sufficient to coordinate expression of genes required to synthesize Da [22]. Such observations are consistent with a simple, perhaps even a one factor–one transmitter code. Acquisition of an appropriate neurotransmitter phenotype often requires coordinated expression of several genes, including enzymes that are essential for the synthesis of transmitters, vesicular transporters, and, in some cases, autoreceptors. Coregulation of such gene cassettes by transcription factors is facilitated in one of two ways: either members of gene cassettes are organized within a single transcriptional unit or operon [23,24] or, when dispersed across the genome, are coordinately regulated by means of common *cis*-regulatory elements [25].

It seems unlikely, even in the relatively simple central nervous system (CNS) of *C. elegans*, that a one factor–one transmitter code is sufficient for all neurotransmitter choices. Indeed, although UNC-3 specifies a cholinergic phenotype in A and B motor neurons [17], all cholinergic neurons are not specified by UNC-3; neither are all UNC-3 neurons cholinergic. For example, UNC-3 is not required for the cholinergic phenotype of the AIY interneuron, which is, instead, governed by the interplay of TTX-3 and CEH-10 [26]. UNC-3 expression is also observed in the noncholinergic ASI chemosensory neuron that releases neuropeptide-like proteins, such as N-acetylneuraminate pyruvate lyase (NPL1) [27,28]. These latter observations support the existence of more complicated and context-specific transcription codes.

Developmental studies in *Drosophila* motor neurons have made important contributions to understanding the mechanisms of neuronal differentiation. Conserved transcription factors, such as Even-skipped (Eve), Islet, Lim3, and Hb9, have been shown to have pivotal roles in neuronal subtype specification [29–32]. These transcription factors are differentially expressed between motor neurons and subsets of interneurons, supporting a concept of combinatorial activity [30,31,33]. Interestingly, it is in interneurons where the potential to specify neurotransmitter phenotypes has been shown. For example, Islet is required for both serotonergic and dopaminergic interneuron phenotypes and, moreover, ectopic expression is sufficient to initiate expression of the Da-synthesizing enzyme tyrosine hydroxylase in some, but not all, neurons. Importantly, ectopic expression must occur early during neuronal development to alter transmitter phenotype, suggestive of the presence of a critical period [33].

In vertebrates, mature NMJs are cholinergic and most, if not all, motor neurons express Islet-1, Islet-2, Lim3 (Lhx3), and Hb9 (MNR2/MNX1), at some stage during their development. Expression of Islet-1, as well as MNR2 and Lhx3, has been associated with a cholinergic phenotype. Thus, ectopic expression of MNR2 in interneurons, normally expressed in paired box 6 (PAX6^+^) motor neuron progenitors, is sufficient to activate a motor neuron-like developmental program including the expression of choline acetyltransferase (ChAT), the rate-limiting enzyme in the synthesis of ACh [18]. However, it is clear that MNR2 (Hb9) alone is insufficient to determine a cholinergic phenotype because it is also expressed in noncholinergic neurons, such as mouse ventral spinal glutamatergic interneurons [34]. Transmitter choice can also be achieved by the active suppression of alternative transmitter phenotypes. Islet-1 participates in an early fate decision between zebrafish primary motor neurons and interneurons by repressing the interneuronal GABAergic phenotype (Figure 1A) and, hence, the motor neuron phenotype is established [19].

Studies of vertebrate interneurons have also provided substantial clues to the complexity of neurotransmitter specification. Interneurons are either inhibitory, expressing GABA or glycine, or excitatory, mostly expressing glutamate. In mouse dorsal horn neurons, two transcription factors, T cell leukemia, homeobox 3 (TLX3) and ladybird homeobox 1 (LBX1), determine whether glutamate or GABA is expressed. Whereas TLX3 promotes the glutamatergic phenotype and suppresses the GABAergic phenotype [35], LBX1 promotes the GABAergic phenotype and suppresses the glutamatergic phenotype [36]. By manipulation of either TLX3 or LBX1, a neuron can be pushed toward excitatory or inhibitory neurotransmission, respectively. In neurons where both TLX3 and LBX1 are coexpressed, TLX3 antagonizes the function of LBX1, thus ensuring that, ultimately, only one of these two transmitters, with diametrically opposite effects, is specified [36].

The above example demonstrates nicely how two transcription factors compete to specify neurotransmitter phenotypes. However, often multiple transcription factors work together to regulate the same neurotransmitter phenotype in different neuronal subsets and, as such, form a combinatorial code. Evidence for combinatorial activity includes the observations that Islet-1 represses the interneuron-specific GABAergic phenotype in zebrafish motor neurons [19]. However, when Islet-1 is misexpressed in GABAergic interneurons, not all go on to acquire a motor neuron identity. Indeed, only interneurons that express the transcription factor Lhx3 seemingly have this potential. Lhx3 is also expressed in motor neurons, suggesting that it is the coexpression of Islet-1 and Lhx3 that facilitates the manifestation of a motor neuron identity [37] and suppression of a GABAergic phenotype [19]. Similarly, in mouse, the GABAergic phenotype in excitatory interneurons is repressed by developing brain homeobox 1 (DBX1) [38]. Intriguingly, both DBX1 and Islet-1 are also expressed in GABAergic interneurons [39–41], indicating that their ability to repress the GABAergic phenotype is context dependent.

Similar to that observed in *C. elegans* [22,26], consensus-binding sequences have also been described for selected vertebrate transcriptional regulators. For example, the mouse ETS domain transcription factor Pet-1 binds a *cis*-regulatory element that directs expression of serotonin pathway genes, including tryptophan hydroxylase 2 (Tph2), the rate-limiting enzyme in the synthesis of serotonin, and solute carrier family 6 (neurotransmitter transporter, serotonin), member 4 (Slc6a4), the serotonin transporter [42]. Moreover, Pet-1 is required throughout development and in to adult life to establish and maintain the serotonergic phenotype. Similarly, the homeodomain transcription factor paired-like homeodomain 3 (Pitx3), together with its interactor Nurr1, regulates genes required for Da synthesis, again through binding to specific promoter elements [43]. These types of observation are consistent with these transcription factors acting as ‘terminal selectors’ of neurotransmitter phenotypes [44].

### Activity-dependent respecification of neurotransmitter phenotype

When considering motor neurons and sensory neurons, a permanent and stable neurotransmitter phenotype might impart stability for function, which would be consistent with the role of these types of neuron. By contrast, within more complex central interneuron networks, neurotransmitter plasticity could offer a mechanism to maintain the important balance between neuronal excitation and inhibition (E/I). An appropriate E/I balance has been hypothesized to be critical for neuronal development, and disturbance has been linked to an increased probability for neurological disorders, such as seizure, autism, and schizophrenia [45]. However, it recently became apparent that both central interneurons and motor neurons can undergo activity-dependent respecification of neurotransmitter phenotype [46,47]. These experiments were carried out in *Xenopus* embryos where different classes of spinal cord neuron show distinct patterns of spontaneous Ca^2+^ spike activity [46]. Experimentally decreasing this activity, by expressing the Kir_2.1_ K^+^ channel, resulted in increased expression of excitatory neurotransmitters, glutamate and ACh, over the inhibitory transmitters, GABA and glycine. By contrast, increasing Ca^2+^ spike activity, by overexpressing a voltage-gated Na^+^ channel, was sufficient to induce a compensatory increase in inhibitory transmitter expression [46]. Such activity-dependent neurotransmitter respecification is achieved through a step in which excitatory and inhibitory neurotransmitters are coexpressed [46], but whether it always results in a complete replacement of transmitters remains to be evaluated (Figure 1B).

Activity can also regulate the occurrence of the neuromodulator serotonin, with increasing activity resulting in a reduction of serotonergic neurons [48]. Where examined, neurotransmitter respecification by activity is transduced via an activity-dependent regulation of transcription factors, such as Tlx3 and Lmx1b [47,48]. As discussed above, in chick and mouse spinal cord, Tlx3 acts as molecular switch that favors glutamatergic over GABAergic neurotransmission. Ectopic expression of Tlx3 is sufficient to increase glutamatergic neurons at the expense of GABAergic cells, whereas loss-of-function mutants show the opposite effect [36]. Neurotransmitter switching, which might be important to maintain an appropriate E/I balance [46,47], is confined to a brief critical period before synapse formation. The details of how electrical activity acts to respecify neurotransmitter phenotype remain to be determined. However, it is clear that activity can alter transmitter phenotype at a time when genetically determined programs were widely believed to predominate.

## Ion channel expression

Just as neurotransmitters underpin neuronal communication within a CNS, so a well-tuned set of voltage-gated ion channels is essential for the ability of the receiving neuron to integrate synaptic information and to instigate an appropriate neuronal output. In contrast to neurotransmitters, the identification of transcription factors capable of regulating ion channel gene expression is more limited. Questions also remain as to whether the same transcription factors coregulate both aspects of neuronal differentiation. As we describe below, early indications suggest that this is indeed so, raising the possibility of coregulation of multiple aspects of neuronal signaling through common developmental mechanisms.

### Ion channel expression, plasticity, and homeostasis

Electrophysiological analysis of mature neurons has generated a paradox. This is because, although it is possible to distinguish, and even identify, neuron subtypes by their characteristic expression of ion channels, the same electrophysiological behavior can, *in silico*, be induced by multiple disparate sets of underlying ion channels [49,50]. Therefore, a key question is the extent to which ion channel gene expression is regulated by developmental, as opposed to activity-dependent, mechanisms. The former might be expected to result in fixed expression levels of ion channel genes, whereas the latter might achieve appropriate circuit outputs through more varied expression patterns.

**Figure 2:**
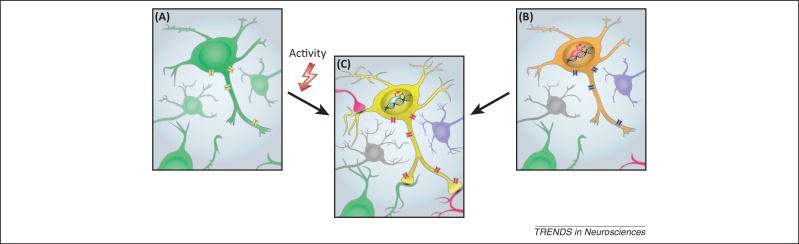
Specification of ion channel expression in embryonic neurons. To acquire the unique electrical properties that will underpin the contribution of a neuron to a circuit, there are two more probable scenarios. **(A)** The neuron expresses a common default set of ion channels, making it almost indistinguishable from other neurons (as indicated by the consistency in color). Once part of a network **(C)**, activity-dependent mechanisms shape the final cocktail of ion channels expressed. **(B)** Individual neuron subtypes express distinct sets of ion channels before the formation of networks, regulated by differential developmental programs (indicated by the different colors of the surrounding neurons). These differences in ion channel repertoire convey distinct functionality to each neuron that might be postulated to reduce the time required to produce functional networks. Similarly, once part of a functional network (C), activity-dependent mechanisms act to fine-tune those electrical properties.

It could be envisaged that the ion channel repertoire of embryonic neuron subtypes is indistinguishable from one another, representing a default, developmentally determined, state (Figure 2). The stereotypic and sequential expression of specific ion channels by developing neurons is consistent with this view [51–53]. By contrast, numerous studies have highlighted the importance of activity-dependent homeostatic regulation of ion channel expression in both developing and mature neurons [54–56]. Where observed, homeostatic regulation of neuronal electrical properties is considered important for network stability by maintaining a constant neuronal output (i.e., action potential firing) in response to changes in network synaptic activity, rapid turnover of ion channels, or unpredictable network perturbations [57]. A key substrate for homeostatic plasticity is the repertoire of voltage-dependent conductances expressed by neurons, in particular voltage-gated Na^+^ conductances [58–62]. Because of the presumed importance that activity has in establishing, refining, and maintaining the ion channel repertoire of a given neuron, the role of developmental transcriptional programs has only very recently been appreciated. Now a new picture emerges in which developmental factors specify neuron subtype-specific ion channel expression profiles before circuit formation. Of course, these properties are likely open to subsequent activity-dependent modification once network activity is established.

### Ion channel specification by developmental factors

Computational modeling has suggested that a fixed neuronal output can be achieved by an almost random combination of many ion channels, creating an almost indefinite parameter space [49]. Electrophysiological analysis of biological neurons shows that the actual parameter space is more restricted. Elegant work from the Marder laboratory and colleagues has shown that the expression of ion channels, in a mature neuron, does not vary randomly but rather in a coordinated fashion, with pairs or sets of channels exhibiting either transcriptional correlation or anticorrelation [63,64]. This may be indicative that, similar to neurotransmitters, ion channels are also regulated in a ‘gene battery’-like manner. Transcription linkage of ion channels might also explain why neurons of the same type or origin can be distinguished by their electrophysiological properties (i.e., by the ion channels they express). In addition, it points toward single neurons expressing a restricted ion channel repertoire that may be acquired through early developmental mechanisms.

Where it has been investigated, ion channel expression, similar to the expression of neurotransmitters, is detected very early during embryonic development and before synapse formation [51,53] (Box 1). It is tempting to speculate that this is because both are coregulated by the same developmental mechanisms. This early expression is independent of external factors, such as synaptic activity, and is seen specifically for ion channels required for basic neuronal excitation, such as voltage-gated Na^+^, Ca^2+^, and K^+^ channels [52]. However, the majority of these studies were confined to mostly unidentified neurons and one cannot exclude the possibility that neurons express a default set of ion channels in a time-dependent manner. What has been missing is a comparative analysis of the acquisition of electrical conductances within individual neurons of particular subtypes (e.g., motor neurons). One of the first model organisms where such a comparative analysis was possible is *Drosophila* due, in greater part, to the stereotypic position of identifiable embryonic motor neurons and their accessibility to electrophysiology.

**Figure 3:**
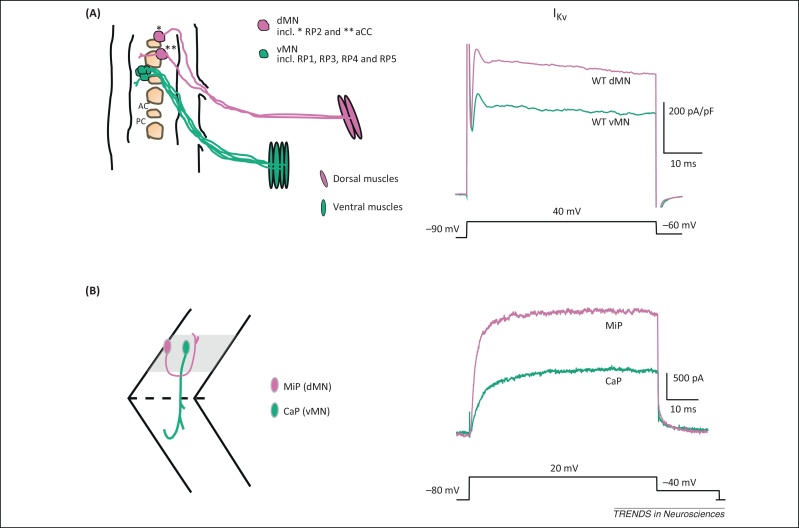
Embryonic motor neurons show distinct electrophysiological properties before active synapse formation**. (A)** Diagram of the ventral nerve cord in a late-stage *Drosophila* embryo. Dorsal motor neurons (dMN, in magenta) express the transcription factor Even-skipped and project to dorsal muscles. Ventral motor neurons (vMN, in green) express the transcription factor Islet and project to ventral muscles. Voltage clamp experiments show differences in outward K^+^ currents between the two motor neuron populations that are independent of synaptic activity. Magnitude of currents are normalized to cell capacitance. **(B)** Diagram of the zebrafish spinal cord depicting two distinct primary motor neurons, the dorsal projecting, Islet-1 expressing MiP (magenta) and the ventral projecting, Islet-2 expressing CaP (green). Voltage clamp recordings show that K^+^ currents are larger in ventral motor neurons compared with dorsal motor neurons. Adapted and modified from [67] (A) and [66] (B).

Within the past few years, it has been established in both *Drosophila* and zebrafish that distinct motor neurons exhibit different electrical properties before synapse formation (Figure 3) [65–67]. These initial studies provided the first clear indications that the expression of at least some ion channels is regulated by early developmental mechanisms and that these are specific to particular neuronal subtypes. In accordance with this hypothesis, ion channels have been identified as potential targets of differentially expressed transcription factors in worms, flies, and vertebrates [17,65,68]. Specific examples include Ca^2+^ and K^+^ voltage-activated channels in *C. elegans* that contain a motif (COE motif), which is recognized by the UNC-3 transcription factor, a determinant of the cholinergic phenotype of type A and B motor neurons [17]. However, no electrophysiological data are available for these motor neurons. By contrast, the terminal selector gene *TTX3* is required for the normal expression of outward K^+^ currents in AIY interneurons [69], although in an earlier study, no specific voltage-gated ion channel target was identified [26]. In *Drosophila* dorsally projecting motor neurons, Eve has been shown to regulate the expression of Slowpoke, a Ca^2+^ and voltage-gated K^+^ channel of the BK family [65]. In addition, Islet-1 knockout was shown to influence the transcription of several ion channel genes in zebrafish motor neurons, as evidenced by microarray studies [68].

**Figure 4:**
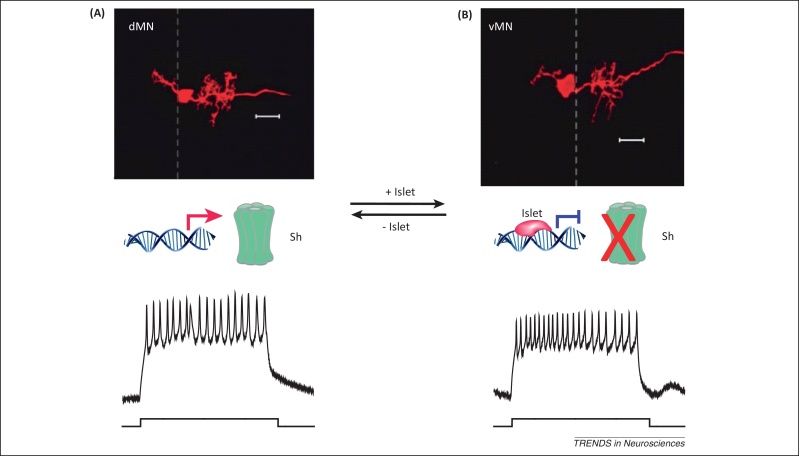
Islet is deterministic for motor neuron subtype electrical properties in *Drosophila*. **(A)** A dorsal (aCC) motor neuron labeled by DiI applied to its neuromuscular junction (NMJ) with its target muscle (muscle 1). The dorsal motor neuron expresses the Shaker K^+^ channel, which conducts a fast activating-inactivating potassium current akin to the A-type current (Box 2, main text). The traces show typical recordings of action potential firing obtained by current injection at the soma (10 pA for 500 ms) via whole-cell current clamp. **(B)** A ventral motor neuron labeled by DiI applied to its NMJ with its target muscle (muscle 6). The ventral motor neurons do not express the Shaker K^+^ channel due to transcriptional repression mediated by Islet. In the absence of Shaker, the neuron fires comparatively more action potentials during 500 ms of 10 pA current injection [compare to (A)]. When Islet is ectopically expressed in dorsal motor neurons, their endogenous Shaker K^+^ current is diminished. By contrast, loss of function of Islet in ventral motor neurons results in a pronounced Shaker K^+^ current [67]. This demonstrates that, at least in *Drosophila*, Islet forms part of an developmental ‘decision-making’ process that is critical to specifying subtype-specific electrical properties in developing motor neurons before neural circuit formation.

Perhaps the most conclusive studies to date for the early developmental regulation of ion channel expression come from *Drosophila*, where a direct link between the differential expression of transcription factors, ion channels, and electrophysiological properties has been recently demonstrated. In the ventral nerve cord of the *Drosophila* larva, motor neurons can be readily identified by cell body position, axonal projection, and muscle targets [70–72]. Differences in axonal targeting, to either dorsal or ventral body wall muscles, have been linked to subtype-specific expression of developmentally important transcription factors, in particular expression of Eve being required for dorsal axon targeting and Islet for ventral axon targeting [29,73]. Whereas Eve is expressed in motor neurons innervating dorsal muscles, so-called ‘dorsal motor neurons’ [29], Islet, Lim3, and Hb9 are expressed in motor neurons that target ventral muscles, termed ‘ventral motor neurons’ [30,32,33]. Recently, it has been shown that dorsal and ventral motor neurons differ in their electrophysiological properties (Figure 3A) [65,67]. Specifically, dorsal motor neurons exhibit larger outward K^+^ currents (Figure 3) and fire fewer action potentials (Figure 4) [67]. Two transcription factors, Eve and Islet, have been linked to subtype-specific ion channel gene expression. In dorsal motor neurons [29] Eve is sufficient to downregulate, but not abolish, the expression of *slowpoke* [65]. By contrast, in ventral motor neurons, Islet is both necessary and sufficient to repress completely the Shaker K^+^ current, an A-type K^+^ current analogous to vertebrate K_v_1.1 [67] (Box 2). Whereas Slowpoke is expressed in both motor neuron types, albeit to varying amounts, Shaker is absent from ventral motor neurons. This is indicative that not only type, but also the relative level, of ion channels are regulated by transcription factors during early development. Such mechanisms would facilitate the acquisition of subtype-specific ion channel repertoires on which plasticity and homeostasis can subsequently act during later postembryonic stages.

## Concluding remarks

Coordinated gene expression, coupled with activity-dependent refinement, underpins the formation of functional neural circuits. The examples described above illustrate the many interactions so far documented between developmental mechanisms (i.e., transcription factors) and neuronal activity in the specification of neurotransmitter phenotype and ion channel expression in developing neurons. Although these are early days, several themes are beginning to emerge that will hopefully be explored over coming years.

The first is that the canonical view of properties such as specification of neurotransmitters being developmentally ‘hard wired’ whereas expression of ion channels is set, largely, as part of activity-dependent feedback (i.e., ‘soft wired’) is blurred by recent experiments. The demonstration that neuron subtype-specific electrical properties are set by developmental mechanisms is particularly important because it is consistent with the viewpoint that neural circuit function and, therefore, behavior, are encoded to some degree within the genome. Although not a new concept [74], these first glimpses of developmental determination of ion channel gene expression offer substantial evidence to support this view. Much might be gained from re-evaluating whether some neurological disorders arise from incorrect developmental specification of ion channel gene expression during early neurogenesis. By contrast, activity-dependent respecification of neurotransmitter content overturns the long-held view that this neuronal property is developmentally fixed. Interesting questions include the precise timing during which the expression of both neurotransmitter-associated and ion channel genes are available to modification during neurogenesis and whether change to one might instigate obligatory change to the other.

A second important theme to emerge is that multiple aspects of neuronal differentiation are regulated by common factors. Perhaps one of the best examples is from *Drosophila*, where the transcription factor Islet is seemingly able to regulate axon guidance, neurotransmitter phenotype, and expression of electrical properties. Might we consider Islet to be a terminal selector and will all of its target genes be identifiable by the presence of conserved *cis*-regulatory motifs similar to what has been observed for some *C. elegans* transcription factors (e.g., ttx-3 and unc-3) [17,22,26]? Related questions include whether there are additional terminal selectors that orchestrate separate or overlapping cassettes of gene targets. Although recent studies in *C. elegans* support this view [75,76], whether this will also hold true for other organisms remains to be determined. Taken to the extreme, this might mean that we will be able to predict key neuronal properties based on the profile of such transcription factor expression.

Although these, and other, important questions remain, the prospect is bright. The recent combination of electrophysiology and molecular genetics, in worms, flies, and fish (to name just a few), offers the prospect of making progress to answer these and other related questions to determine the relative contribution of developmental versus activity-dependent mechanisms for the formation of neural circuits.

## Figures and Tables

**Figure I:**
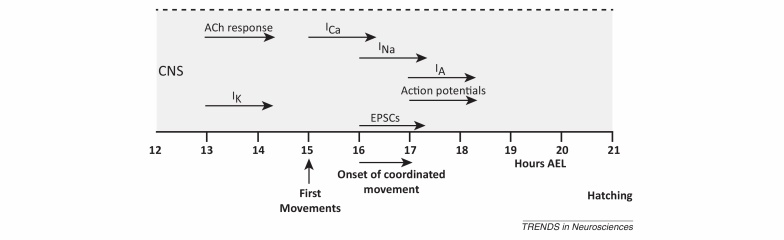
Time line for development of motor neuron electrical properties. Abbreviations: ACh, acetylcholine; AEL, after egg laying; CNS, central nervous system. Adapted from [51].

## Box 1 Development of electrical properties of Drosophila embryonic central neurons

The first appearance of each current, recorded in motor neurons, and its continuity throughout development are shown in Figure I. Important behavioral outputs, such as first muscle movements to hatching, are also indicated. Time is counted from initial egg laying in hours.

The first currents to express are the voltage-activated delayed rectifier K^+^ conductance and ligand-gated ACh currents. These are followed by voltage-activated Ca^2+^ and Na^+^ currents. A-type K^+^ currents, including Shaker and Shal, coincide with the appearance of the first action potentials, which are preceded in these neurons by excitatory postsynaptic potentials (EPSCs). Ionic currents similarly develop in a time-dependent manner in *Xenopus* neurons; however, here, voltage-gated Ca^2+^ currents precede other currents [53]. Similar to *Drosophila*, the A-type K^+^ current shows a late onset in *Xenopus* [52].
